# Parental Perceptions of a Manchester Service for Autistic Spectrum Disorders

**DOI:** 10.1155/2011/601979

**Published:** 2011-06-19

**Authors:** Mischa Mockett, Jamila Khan, Louise Theodosiou

**Affiliations:** ^1^Child and Adolescent Psychiatry, The Winnicott Centre, Manchester M13 0JE, UK; ^2^University of Manchester, Manchester M13 9PL, UK

## Abstract

*Background*. User feedback is now an integral part of both clinical governance and service development, and it also provides a key route to engaging parents and children. Autistic spectrum disorders (ASDs) can impact on all members of a family, and close working between parents and professionals is essential. *Aim*. To explore parental satisfaction rates and identify areas in need of improvement. *Method*. A postal survey was completed by parents whose children had been diagnosed with an ASD in the past 18 months in a Manchester Child and Adolescent Mental Health Service. The National Autism Plan for Children was used as a gold standard. *Results.* Parents were particularly satisfied with the way team members dealt with them and their children during appointments. However, the standard of written information provided about the condition, diagnosis, and support available could be improved. The findings show the benefits of receiving a diagnosis in the recommended timeframe. *Discussion*. We discuss ways of effectively using scarce resources.

## 1. Introduction

Autistic spectrum disorders (ASDs) are developmental disorders estimated to affect 1 in 150 children [1]. They are characterised by social communication impairments, limited imagination, and repetitive behaviours. Learning difficulties are overrepresented, but not always present. Increasing professional awareness has led to higher levels of diagnosis and support, possibly explaining the recorded rise in prevalence over the last 30 years [2]. ASD is commoner in males (M : F autism 4 : 1, aspergers 10 : 1). Genetic factors are complex, and no single gene mutation or chromosome abnormality has been linked [3], although the condition has the highest heritability of any psychiatric disorder (approximately 90%) [4]; the risk of having a second child with autism is increased by 20–30. 

The National Autism Plan for Children (NAPC) emphasises the importance of early intervention and close collaboration between parents and professionals and provides a gold standard for services [3]. Benefits for parents include increased capacity to seek information from external sources and make use of available health services [3]. 

Diagnosis of children with ASDs requires multiagency and multidisciplinary collaboration. In 1998, an assessment team was established in one sector of Manchester. This service involved sessions from child and adolescent psychiatrists (CAP), community paediatricians (CP), CAMHS practitioners, specialist speech and language therapists (SALT), and educational psychologists (EP). The successful format was established city wide in 2000. The multiagency teams are called Social Communication Assessment and Intervention Teams (SCAITs) [5]. Service provision within CAMHS needs to make the best use of limited resources [5]. 

In addition to the core symptoms, there are other common features [6], such as abnormalities in sleep and mood. For a diagnosis of ASDs to be made, there must be both symptomatology and an impact on functioning [3]. If there are concerns during developmental screens, specific screening for autism is strongly encouraged. Parental concerns provide vital information for early diagnosis [3]. Specific diagnostic instruments are available and should be used alongside clinical judgement. Most commonly used is the Autism Diagnostic Observation Schedule (ADOS) [3]; however not all characteristics may be demonstrated during the test. Observations should be conducted in different settings including clinics, homes, and schools.

ASD is a developmental disorder; symptoms can vary in severity at different times. Difficulties can become more apparent when changes in routine occur, for example, starting a new school, and this is often when children are referred. The benefits of early identification of ASDs are recognised by parents and professionals alike [3]. The diagnosis of autism, however, is often not made until 2 to 3 years after symptoms are recognized [3]. Evidence suggests that early intervention can lead to improved outcomes for most children [7]. Earlier diagnosis facilitates earlier educational, social, and medical support. It is also important to reduce waiting times from referral to intervention. There are considerable demands on parents as they accept and adjust to their child's communication and social interaction impairments [3]. 

Many people seek information on their own, from other parents, websites, books, and autism groups and newsletters [3]. Children and families want accessible mental health services which provide support when needed and involve them as users. They also want to know what services are available to help them support their child [3]. 

The NAPC guidelines address identification, assessment, diagnosis, and access to early interventions for children with ASDs. It recommends a three-stage assessment framework. Stage 1 is a general multidisciplinary developmental assessment for any child with possible developmental problems. Stage 2 is a multiagency assessment. The assessment should be completed and fed back to the family within 17 weeks of referral. A written report should be produced and discussed with the parents. Stage 3 addresses the need for tertiary referral. The local team may need this for reasons such as specific advice about treatments. The NAPC states that feedback to parents should include information about services, for example, The National Autistic Society (NAS).

In Manchester, all SCAIT professionals receive training in assessing ASDs. Monthly SCAIT meetings involve liaison work with community paediatricians and preschool special needs workers. Direct referrals are accepted from general practitioners and paediatricians. Referrals from educational psychologists, health visitors, and school nurses are accepted if the general practitioner or a paediatrician is informed. The team generate a plan tailored to each individual regarding diagnosis and care.

Parent intervention programmes can enhance interaction with children, promote development, and increase parental satisfaction and mental health [3]. SCAIT offers five 2-hour sessions: Understanding ASD, Understanding and managing behaviour, Understanding and working with the education system, Enabling your child's communication and understanding, and Further resources for you and your child.

## 2. Aim

To assess parental satisfaction with SCAIT assessments and compare the service with the NAPC Guidelines. 

## 3. Method

Parents and carers of the 35 children diagnosed with ASDs from December 2008 to May 2010 were invited to participate. Questionnaires and a covering letter were sent out with a self-addressed envelope. Participants were invited to have a telephone or face-to-face alternative to complete the questionnaire. The work was undertaken as an audit, thus ethical approval was not needed.

A previous Manchester audit tool was enhanced to provide further information about the postdiagnostic group (Appendix 1). Questionnaires were scored according to total satisfaction, and satisfaction in each of the 4 areas of the assessment process. Compliance with the NAPC Guidelines was also recorded.

## 4. Results

**Figure 1:**
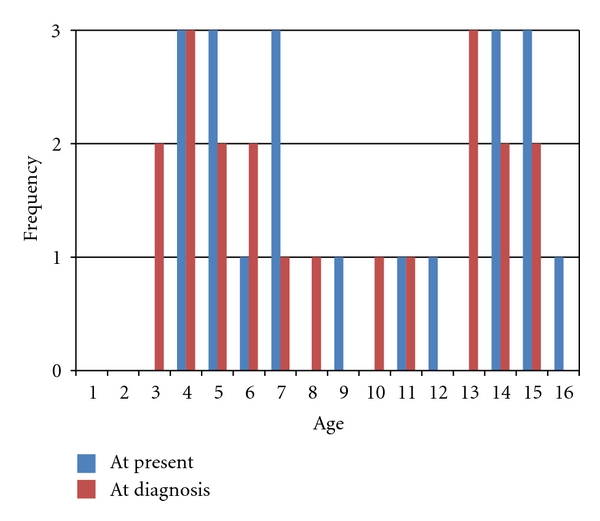
Ages of children participating in audit.

Thirty-five questionnaires were posted, and 20 (57%) parents participated, with 1 father responding. Of the 20 participants, 1 parent completed the interview via telephone and the other 19 returned the questionnaire by post. The child's age at diagnosis and the present time showed 2 peaks (Figure 1), consistent with transition to primary and secondary school.

Most parents (86%) were especially satisfied with the assessment process (Table 1), but less happy with the information received before and after the assessment. 

Most parents felt they were well informed about the assessment before the appointment, with 65% receiving information. However, 14 would have liked to have known the different parts of the process, and 8 wanted to know the questions they would be asked. About half of parents would have liked to have known the name and professional background of the clinician (Table 2).

Nineteen parents felt they were definitely listened to carefully, with the final patient choosing yes to some extent. Additionally, 18 parents felt they were able to discuss their concerns and give feedback at the time of assessment, and 80% felt the assessment could not have been communicated differently. The family who needed an interpreter reported that the information they were provided with before the appointment was very poor, although they believed the actual process was good.

Notably, 85% of parents would have liked a letter with a plan for further assessment and appointment dates. Most parents had received a report which 65% claimed to definitely understand, with 20% understanding to some extent. Parents would have liked less medical terminology in the reports. Eighteen parents had the opportunity to give feedback at the time of the assessment. However, 59% stated they either did not have any say or only to some extent. All of the parents said they were able to ask questions at following meetings.

With regards to the postdiagnostic workshops, 8 parents received information regarding the aims. When parents are invited to attend the sessions, written information about the course is also sent with the letter. However, the 8 who did not answer may not have been invited.

**Figure 2:**
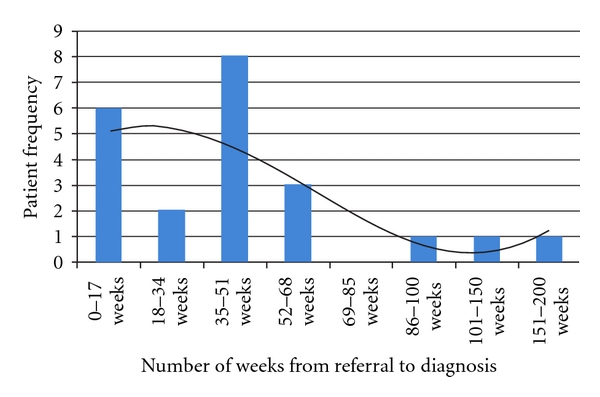
Time period between referral and diagnosis.

Every respondent was happy with the assessment process, with 60% reporting it to be very good, 100% of parents said they were definitely treated with trust and dignity. After the assessment, 100% of patients were happy with the level of information they had regarding the process. Although 18 parents received additional information in some form, 6 felt they had not received enough information about the condition and future interventions. With regards to the postdiagnostic workshops, only 15 parents said they were invited to attend. The reasons for this were not explored, although one patient did report moving house. When asked why they were unable to attend, Childcare was a key theme as was timing. Looking at the time interval from referral to diagnosis, great variance was apparent (Figure 2). The average time was 46 weeks with only 6 diagnoses reported in the recommended time frame of 17 weeks.

Of note, the child with the shortest timeframe from referral to diagnosis was the least satisfied, while the longest timeframe elicited the highest satisfaction score. The results show 2 peaks, with satisfaction higher at the times of school transition (3–5 and 10–12 year olds). 

## 5. Discussion 

One weakness was size, although sampling the past 18 months allowed the collection to be achieved within the allocated timescale. If the study was increased to the past 3 years, the results may show a more significant trend, although parents might find the process harder to recall. One solution might be administering the questionnaire as a standard part of clinical practice.

Parents wanted more information regarding diagnosis and management. A longer follow-up appointment after diagnostic feedback could allow needs to be individually assessed. During this appointment, professionals could discuss the purpose of the postdiagnostic workshops. The information provided during these workshops is obviously most beneficial when attending the group sessions, as they are interactive and parents have the opportunity to share experiences and strategies. However, if all parents received the written information provided within these sessions, it would be helpful to those who could not attend. As childcare and timing were the commonest reasons for nonattendance, this could be addressed by combining all 5 sessions into 2 days within school hours.

Written communication could be enhanced by a standardised response letter from the SCAIT team. This could include the role of SCAIT, the purpose of the assessment, the appointment time, the assessment structure and timescale, the name and profession of all clinicians, and possible question areas. This should help ease parent's anxiety and ensure that scarce appointments achieve maximum benefit. Finally, the assessment report needs to be clear and concise. The initial page should contain a standardised format with the outcome clearly stated, the individuals and professional roles of those involved, and the date of the diagnosis. 

Although, most parents were happy with the assessment service, satisfaction could be increased by providing more information about the assessment as well as the condition. It is important to actively involve parents in the sessions, allowing them to build a trusting relationship with the health professionals involved as children may need support from the team for many years. Giving each family the time to discuss their concerns will also help tailor individual needs and further increase parental satisfaction. The fact that parents are considerably more likely to have a second child with ASDs emphasises the importance of engaging families.

The study was fed back to SCAIT, and standardized letters and workshop information sheets have already been implemented. Furthermore, the team will complete the audit loop by undertaking the audit again in 18 months time. This study has provided a key opportunity to offer a value-added service with service-user feedback. 

## Figures and Tables

**Table 1:** Average satisfaction rates with the assessment process.

Satisfaction with entire process	Satisfaction before the assessment	Satisfaction with assessment process	Satisfaction with assessment outcome	Satisfaction with postdiagnostic workshops
71%	63%	86%	62%	57%

**Table 2:** Information provided before the service.

How well were you informed about the assessment before the first appointment?	Very good	6
	Good	11
	Average	1
	Poor	0
	Very poor	2

Were you given information describing the assessment process before the appointment?	Yes	13
	No	7

What information would have been helpful before seeing the clinician? (choose all applicable options)	Name and profession	1
	Questions to be asked	1
	Time it would take	1
	Different assessment parts	4
	Not answered	3
	Time it would take and different parts of the assessment	1
	Name and profession and different assessment parts	2
	Questions to be asked and different assessment parts	5
	Name and profession, questions to be asked, and different assessment parts	2

Did you know of the name and professional background of the clinician prior to attendance?	Yes	9
	No	10
	Not answered	1

If you did not know the name and professional background would you have liked to?	Yes	12
	Not answered	8

**Table 3:** The assessment process.

Ease of getting to CAMHS?	Very good	10
	Good	6
	Average	2
	Poor	1
	Very poor	1

Which CAMHS professionals did you see for assessment (not intervention)	CAP	8
	CP	3
	SALT	2
	Child mental health practitioner	1
	CAP and SALT	1
	CP and SALT	1
	CAP and CP	3
	CAP, CP, and SALT	1

**Table 4:** Relationship with the professional performing the assessment.

Did the professional listen carefully to you?	Yes definitely	19
	Yes to some extent	1
	No	0

Did you have trust and confidence in the professional you saw?	Yes definitely	15
	Yes to some extent	5
	No	0

Were you treated with trust and dignity?	Yes definitely	20
	Yes to some extent	0
	No	0

Were you given enough time to discuss your concerns about your child?	Yes definitely	18
	Yes to some extent	2
	No	0

Could the communication have been done differently?	No	16
	Yes to some extent	3
	Yes definitely	1

**Table 5:** Outcome of the assessment.

After the first meeting would you have liked a letter with the plan for further assessment and appointment dates?	Yes	17
	No	2
	Not answered	1

Were you given the opportunity to provide feedback at the time of the assessment?	Yes	18
	No	2

How satisfactory was the assessment process?	Very good	12
	Good	8
	Average	0
	Poor	0
	Very poor	0

Was there part of the assessment process you would have liked to have been done differently?	Yes	2
	No	18

**Table 6:** Written information.

At the end of the assessment did you have enough information regarding the process?	Very good	8
	Good	19
	Average	3
	Poor	0
	Very poor	0

How was the assessment outcome communicated?	Verbally only	3
	Written only	1
	Written and verbal	16

Could the assessment have been communicated differently?	No	15
	Yes to some extent	3
	Yes definitely	2

At the end of the assessment were you given/posted a report?	Yes	17
	No	3

Did you understand the report?	Yes definitely	13
	Yes to some extent	4
	No	2
	Not answered	1

Did the report contain an initial page with the outcome of the assessment clearly documented?	Yes	16
	No	2
	Not answered	2

Were you able to discuss the report at the next appointment?	Yes definitely	12
	Yes to some extent	5
	No	2
	Not answered	1

Did you have a say in what the report should contain?	Yes definitely	7
	Yes to some extent	5
	No	6
	Not answered	2

Were you given a chance to ask questions either in the feedback or following meeting?	Yes definitely	15
	Yes to some extent	2
	No	2

**Table 7:** Additional information.

Did you receive additional information regarding your child's condition at the end of the assessment?	Yes verbally	2
	Yes the written report	1
	Information from early support	1
	NAS leaflets	1
	Multiagency partnership (MAP) leaflets	0
	Not answered	2
	Verbally and written report	1
	Verbally and NAS leaflets	2
	NAS and MAP leaflets	4
	Verbally, NAS and MAP leaflets	2
	Verbally, information from early support, and NAS and MAP leaflets	1
	Verbally, the written report, and NAS and MAP leaflets	2
	Verbally, the written report, early support, and NAS and MAP leaflets	1

If MAP was recommended to you, were you happy for your child to be referred?	Yes definitely	14
	Yes to some extent	0
	No	3
	Not answered	3

Did you receive enough information about the condition itself and future interventions?	Yes	14
	No	6

Were you given the opportunity to watch a video about the condition following diagnosis?	yes	4
	No	15
	Not answered	1

**Table 8:** Postdiagnostic workshops.

After diagnosis were you invited to attend the Autism Workshops?	Yes	15
	No	3
	Not answered	2

Were you able to attend the sessions?	Yes	8
	No	7
	Not answered	5

If you were unable to attend, which reason best applies to you?	Inconvenient location	3
	Inconvenient time	1
	No transport	0
	No childcare	3
	Other	3
	Inconvenient location, no transport or childcare	1
	Not answered	9

Were you given any information regarding the aims of the course?	Yes	10
	No	2
	Not answered	8

Did the aims of the course appeal to you?	Yes	9
	No	2
	Not answered	9

Was the course useful?	Yes	8
	No	2
	Not answered	10

Did the course cover what you wanted to know?	Yes	9
	No	0
	Not answered	11

Would you recommend the course to other parents/carers?	Yes	9
	No	0
	Not answered	11
